# Shouldering the Burden of Evidence-Based Practice: The Experiences of Physiotherapists Partaking in a Community of Practice

**DOI:** 10.1155/2016/9051378

**Published:** 2016-01-24

**Authors:** Karen McCreesh, Louise Larkin, Jeremy Lewis

**Affiliations:** ^1^Department of Clinical Therapies, University of Limerick, Limerick, Ireland; ^2^Department of Allied Health Professions and Midwifery, University of Hertfordshire, Hatfield, Hertfordshire AL10 9AB, UK; ^3^Musculoskeletal Services, Central London Community Healthcare NHS Trust, London SW1E 6QP, UK

## Abstract

The study aim was to elicit the motivators, barriers, and benefits of participation in a Community of Practice (CoP) for primary care physiotherapists. We used a qualitative approach using semistructured interviews. The participants were twelve physiotherapists partaking in a newly formed Shoulder CoP. A desire for peer support was the strongest motivator for joining, with improving clinical practice being less apparent. Barriers to participation included time and work pressures and poor research skills. The structure of the CoP, in terms of access to meetings and the provision of preparation work and deadlines for the journal clubs, was reported to be a facilitator. Multiple benefits ensued from participation. The role of teamwork was emphasised in relation to reducing isolation and achieving goals. The majority of participants reported positive clinical practice changes in terms of improved patient education, increased confidence, and availability of new resources. All participants reported some element of personal growth and development, in particular in their evidence-based practice skills. The results provide support for the use of CoPs as a means of continuing professional development for physiotherapists in the workplace, as significant benefits are gained in terms of evidence-based practice (EBP), patient care, and therapist personal development.

## 1. Introduction

Healthcare professionals, including physiotherapists, are experiencing increased demands to use research evidence in clinical practice [1]. A recent systematic review of EBP in physiotherapy highlighted multiple barriers, including time and workload pressures, limited access to research literature, poor skills, and perceived mismatch between research and practice [2]. Interventions to improve EBP were also reviewed, with journal clubs and knowledge broker interventions showing the best effectiveness. Bridges et al. [3] surveyed over 900 physiotherapists regarding influences on their propensity to adopt EBP and concluded that multiple strategies would be required to effect change in clinical practice.

The term Community of Practice (CoP) was developed by Lave and Wenger [4] to describe learning through practice and participation in groups. Lave and Wenger's initial interest was in how apprentices learn. However, the CoP concept evolved and has come to be used as an intervention tool in a wide range of domains. Communities of practice are “groups of people who share a concern, a set of problems, or a passion about a topic and who deepen their knowledge and expertise in this area by interacting on an ongoing basis” [5]. It is clear that this definition encompasses many entities that are common to everyday clinical practice, such as multidisciplinary teams or clinical interest groups. For many of these groups, learning is incidental to the group's interaction, while for others learning is the reason they have come together. The characteristics of a CoP are identified as “domain,” the common ground shared by members; “community,” the structure that facilitates member's interactions; and “practice,” the specific knowledge, skills, and resources shared by members. The CoP is a learning concept which emphasises situational learning within the practice environment and encourages mutual engagement, joint enterprise, and a shared repertoire of resources. Li et al. [6] examined how CoPs were utilised in business and healthcare sectors, as well as their effectiveness in promoting the best practice. They describe four dominant characteristics of CoPs in these sectors, which are social interaction among members, knowledge sharing, knowledge creation, and identity building. Ranmuthugala et al. [7] reviewed how and why CoPs are established in healthcare. They noted a shift from focus of CoPs on exchanging information and knowledge, towards more recent research where CoPs were used as a tool to facilitate the implementation of EBP. They also described the challenges in evaluating the effectiveness of CoPs due to the complex and multifaceted interventions involved.

While many studies have described the use of CoPs in the health professions [7], there is limited research describing the use of CoPs in physiotherapy. Evans et al. [8] described the discussion board contributions of physiotherapists to an online continuing education programme framed in CoP learning principles, suggesting that the model promoted the creation and sharing of new knowledge amongst participants. Wilding et al. [9] described a year-long CoP action research project amongst occupational therapists (OTs) in Australia and examined the experiences of members. Two major themes emerged: firstly, promotion of scholarship, which describes how participants began to think more critically about practice, and secondly, promoting professional confidence, passion, and cohesion, where peer support fostered increased confidence in the OTs own practice. It is clear that the CoP model is a potentially valuable framework for the development of practice-based learning in healthcare settings, including physiotherapy. This study describes the formation of a new CoP for physiotherapists working in primary care, focused on the management of shoulder pain. Based on the dearth of relevant research concerning CoPs in physiotherapy, the purpose of the study was to examine the experiences of physiotherapists taking part in a CoP.

## 2. Methods

### 2.1. Participants

The Shoulder Community of Practice Project was developed as a knowledge exchange and dissemination initiative to improve clinical practice in the management of shoulder pain in public primary care physiotherapy settings in Ireland. Based on the growing evidence for its usefulness as a practice-based learning tool, the CoP model was determined to be the best framework in which to develop the project. The project involved a number of components and ran over a 9-month period, led by a physiotherapy academic, who was undertaking research in the area of shoulder pain (KM). See the Shoulder CoP Project Description for a description of the structure and activities of the CoP. By the end of the 9-month CoP project period, the group had met seven times, reviewed 25 papers in six journal clubs, developed and launched a website, and developed and implemented a protocol for shoulder exercise classes. All Shoulder CoP participants were invited to take part in semistructured interviews to explore their experiences of participating in the CoP. There were 16 members who engaged with the CoP over the 9-month period; of these, 12 consented and were available for interview.

*The Shoulder CoP Project Description.* The Shoulder Community of Practice (CoP) project involved the following:

- One-day shoulder pain seminar was conducted (attended by 120 physiotherapists). Shoulder CoP was introduced and physiotherapists were invited to join (the aim was for 15–20 members; inclusion criteria were as follows: physiotherapists, primary care caseload, and shoulder pain forms at least 10% of caseload). World Café-style event was used to elicit attendees' priorities and ideas for a Shoulder CoP, which included journal clubs, development of patient and practitioner resources, and discussion groups/peer support.
- Sixteen eligible physiotherapists joined the CoP. Objective-setting exercise undertaken with the group, based on World Café data and members own needs, determined the CoP plan which included monthly journal clubs, a clinical practice project, and a website.
- A website was developed in parallel with the CoP meetings (http://www.shouldercommunity.com/) and used to disseminate the work of the CoP to the broader physiotherapy and public community.
- Meetings, which participants could attend either face-to-face or via teleconference, were held on a monthly basis. Mean meeting attendance across the 7 meetings was 70%. The CoP leader (KM) acted as meeting Chair, facilitated discussions, and acted as content expert as required throughout the process. A research assistant (LL) undertook all CoP administration tasks (e.g., organising meetings, minute-taking, and group communication).
- Monthly journal clubs were themed around areas of interest, for example, exercise in shoulder pain and outcome measures. A range of papers were chosen from member and CoP leader suggestions. Papers were reviewed by members in small groups and a joint summary of each paper was produced. Support was given for critical appraisal using critical appraisal tools or tutorials provided by a physiotherapy academic (KM) at the early stages, with less support later in the process.
- Clinical project was developed after 3 months of the CoP. Based on journal club findings and clinical needs, a protocol was developed for group shoulder exercise classes through discussion and group feedback. These classes were implemented in local settings by the physiotherapists over the subsequent months, with CoP meetings used to provide peer support for this initiative.

### 2.2. Design

The study method was a broad qualitative approach, with the aim of gathering rich data about the participants' own experiences; therefore, individual interviews were deemed the most appropriate method of data collection. Semistructured interviews were conducted by telephone by a research assistant. The question route was devised based on the aims of the research, beginning with an open question and later asking for specific examples (see Interview Question Route). The duration of interview was approximately 25 minutes. Interviews were audio-recorded and transcribed verbatim. Transcripts were sent to each participant for confirmation. Ethical approval for the study was received from the University Ethics Committee, and all participants gave written informed consent.

*Interview Question Route.* Semistructured Interview Question Route was as follows:

- Tell me about your experience of the Community of Practice (CoP).
- Why did you decide to join the Shoulder CoP?
- Tell me what challenges you think there are for participating in the Shoulder CoP.
- Tell me what benefits you think there are for participating in the Shoulder CoP.
- Can you describe a situation where being part of the Shoulder CoP has impacted your clinical practice?
- Can you suggest modifications to the CoP so that it is used to support clinical practice?

### 2.3. Data Analysis

Thematic analysis was used for data analysis, using the six-stage guide as described by Braun and Clark [10], as it offers the potential to provide rich and detailed account of the data. Two investigators independently undertook data analysis (KM and LL), both of whom were closely involved in the activities of the CoP, KM as the project leader and LL as a research assistant, who undertook administrative activities to support the CoP. A qualitative data analysis package NVivo (V10) was used to assist in performing data coding and forming initial categories. We took an inductive approach, where the thematic analysis was data driven and explorative rather than from an existing theoretical framework. The first stage of analysis consisted of familiarisation with the data through repeated reading. Initial coding was then undertaken, with further refinement into potential themes. At this stage, the two investigators reviewed and discussed the themes generated, and finally, consensus was reached on the naming and definition of themes, and data extracts were chosen to illustrate meaning of each theme. A limited degree of triangulation was carried out by comparing the interview themes with the minutes of the final CoP project meeting, where all members attended in person to discuss their experience of the project and to plan for future development of the CoP. Individual interviews had been carried out prior to this meeting.

## 3. Results

**Figure 1:**
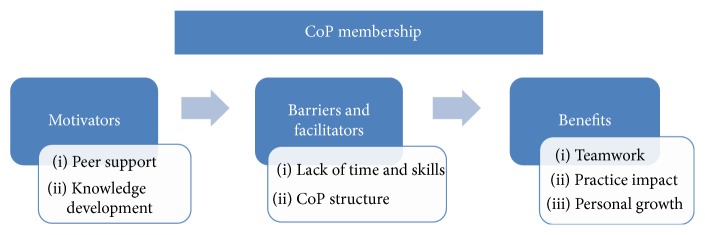
Themes identified from the interviews.

Twelve physiotherapists (2 males and 10 females) took part in the study. They had an average of 12.7 years of practice experience, with eight working wholly in a primary care setting and four in both primary and acute care settings. The main themes identified from interviewee responses were “motivation,” “barriers and facilitators,” and “benefits of the CoP.” The findings within the “barriers and facilitators” and “benefits” themes were strongly supported by comparison with the minutes of the final CoP project meeting. The themes are represented graphically in Figure 1.

### 3.1. Motivation

As one of the opening questions to the interviews was related to what motivated the therapists to join the CoP, it was unsurprising that motivation emerged as a strong theme in the early part of the interviews. For the majority of participants (9), the motivation to join the CoP was driven out of a sense of being isolated from other physiotherapists in their daily practice and seeing the CoP as a way of gaining peer support and more interaction with colleagues.

*PT06: I decided to join because … I work in a very small practice and I felt that it was a way of interacting with other physiotherapists and other people with expertise different to my own.*

*PT03: I thought it would be quite a nice thing just to have a community out there that you could access particularly when I work on my own. I'm in a health centre so I mightn't always have someone I can bounce ideas off.*

A desire to improve their clinical practice, or to address perceived gaps in knowledge in the area of managing patients with shoulder pain, was less apparent in the interviews. Only a small number of participants (4) made statements such as the following.

*PT08: I joined to increase my knowledge and improve my quality of care.*

*PT05: For years I'd never been happy with shoulders … so that's why I joined it really to help progress my knowledge, I suppose and help improve the outcome for the patients that I see with shoulder problems.*

### 3.2. Barriers and Facilitators

The themes of barriers and facilitators to involvement in the CoP were extensive throughout the data. Since there was overlap between similar factors being considered barriers and facilitators, both are discussed under the same theme heading. “Time” was the main barrier mentioned, while “EBP skills” and the “organisation of the CoP” emerged as both barriers and facilitators.

#### 3.2.1. Time

All of the participants cited time pressures, both work and personal, as a barrier to their involvement in the CoP. Many (8) also said that they conducted some CoP activities, for example, reading journal articles, outside their paid working hours.

*PT09: The time aspect, that you do need to invest some of your own time in reviewing the literature and then some work time for the meetings. So that was definitely a bit of a barrier. *

Time was also mentioned as a factor in terms of gaining permission from line managers to participate in the CoP, with three physiotherapists describing how they had to convince their manager of the benefits of their involvement.

*PT02: I suppose I justify it by if I can get better at treating (shoulder patients) they'll get out the door quicker and it'll be more effective, so my manager had no problem with it.*

*PT11: Within the (health service) when you go to a meeting like that well our boss wants to show what the department is gaining from you going to the meeting … so there's that challenge I suppose when you are in … the public sector with regard to using your time wisely.*

#### 3.2.2. Skills

A smaller number of therapists (5) commented that their lack of skills in reading and reviewing journal articles was a barrier to full participation in the journal club at the initial stage. However, comments from others indicated that participants developed skills in reading, understanding, appraising, and summarising as the meetings progressed. The following comments indicate that participation in journal club activities facilitated learning.

*PT05: Well when I was reading the journals I have absolutely no background in statistics … I've never done statistics so that would have been a major problem when I was reading these papers. *

*PT04: Maybe a lack of familiarity with some of the research methodologies and that probably was a challenge, but again that's something that can be embraced and learned out of as well.*

#### 3.2.3. Organisation of the CoP

In the main, the organisation of the CoP was seen as a facilitator to participation. While undertaking work for the journal clubs was seen as a challenge for some, physiotherapists appreciated the provision of preparation work before the journal clubs, along with timelines to complete tasks, which provided an impetus to undertake activities that might otherwise not be prioritised as part of a busy clinical workload.

*PT06: I suppose a challenge is doing the assignments or whatever project you have on a given month but I think that's something that you should expect, you shouldn't get information handed to you because I don't think you take it in as much anyway.*

*PT11: Because you had deadlines and dates and things it made you do things that you wanted to do but you don't get around to doing, so that would give you an opportunity or reason for making sure you did those things.*

The opportunity to access CoP meetings by teleconference was seen as positive by most participants (8); however, two suggested that face-to-face meetings provided additional benefits in terms of personal interaction and providing protected time to engage.

*PT10: I thought it was very accessible. I loved that we had the teleconferences because I'm coming from (distant site). There was always no issue with me using the teleconference you know rather than being there in person and I thought they worked really, really well.*

*T05: The first day I did the telephone thing but I just found it easier when I went to the meetings rather than doing the teleconferencing because again I can't do that at work, I have to do it at home … I think if I go to the meetings I preferred that because then I couldn't be interrupted and I could keep that time for that.*

### 3.3. Benefits of the CoP

Each of the participants cited multiple benefits of involvement in the CoP, which were categorised under teamwork, clinical practice impact, and personal growth.

#### 3.3.1. Teamwork

The CoP was reported by all participants to provide peer support, which helped reduce the isolation of those working alone or isolated from colleagues. There was also a sense of shared interests in the area of shoulder pain. Therapists also valued the variety of experience and opinions brought about by having CoP participants from different practice settings.

*PT03: I think you know being part of a group of people who can contact each other and communicate with each other about different shoulder conditions is a really handy thing to do, particularly working as a lone physio in a health centre.*

*PT07: It linked up people across different settings and allowed a good sharing of information without any judgement.*

Six out of the 12 participants discussed how more was achieved as part of the group than could have been accomplished alone, in terms of amount of information that could be evaluated, but also how others could add different opinions or perspectives.

*PT04: It certainly was more enjoyable than sitting down reading articles on your own and I think that's kind of an important kind of factor of the Community of Practice, that it is a community as opposed to you sitting at home doing it all yourself and it does break the workload.*

*PT09: I suppose kind of working together with a few people at the one time trying to produce one document was good because you're taking other people's views into consideration, so that was useful rather than just critiquing it on your own.*

#### 3.3.2. Clinical Practice Impact

More than half of the participants (7) mentioned having increased confidence in their clinical practice that came from enhanced knowledge. Physiotherapists reported improvement particularly in their education of patients and in their choice of evidence-based treatment options.

*PT06: I'm inclined to educate the patients early on in their treatment so that they are aware of all the options and what the current research says about each option. So I definitely have changed my practice with them.*

*PT12: I suppose one of the biggest things really would be that I'd be more focused on using outcome measures because we've appraised the articles on the outcome measures and had a look at the evidence base behind those.*

For the more expert physiotherapists in the group, clinical practice impact came not so much in terms of practice change, but in reinforcement of existing practice.

*PT10: Maybe I'll have a bit more confidence with discussing cases with consultants having had that reinforcement as a group, you know condensing that knowledge.*

While the majority of physiotherapists mentioned some positive impact on their clinical practice, one dissented, describing how the CoP was too strongly focused on research, and did not have enough of a clinical focus to meet their needs.

*PT08: At the start it was very evidence-based and a lot of it was very theory-driven … it was very research-based. Clinically I felt it was lacking … I would have thought maybe that there would have been maybe a lot more discussion about … troublesome patients.*

This was in contrast to the views of another participant who found that the CoP was more clinically relevant than traditional CPD activities.

*PT01: I found it I suppose very practical, very relevant to what we're doing here in our day-to-day clinic, which I find some courses and stuff I go to aren't … You know I certainly feel I've come away with something concrete in my hand and something that I'm using almost day-to-day in my clinical practice.*

The projects of the CoP were also cited by the participants as having a positive impact on their day-to-day clinical practice. The CoP website was reported to provide an evidence-based resource to which patients could be directed.

*PT03: I had a patient today with a tear of his rotator cuff so I'll send him on to (the CoP website) and he'll be able to get a lot from it because he's quite big on IT and I know he's Googled his problem, but I'd rather direct him to something that's evidence-based than him just to be reading anything that's on Google.*

Three participants highlighted their involvement in the clinical project that is running shoulder exercise classes, as having significant impact on their clinical practice.

*PT04: Probably the development of the classes has been probably the big one in terms of my practice … you know that (the patients) can go to a class and you can discharge them a little bit sooner … because you know that they'll be caught, asked if there's any difficulties. So that's probably the main change in my practice related to that.*

#### 3.3.3. Personal Growth

The physiotherapists described several areas of personal growth and development that resulted from their involvement in the CoP. These included a sense of having been challenged (8), followed by a feeling of achievement in seeing their skills improve.

*PT03: Well the meetings I think they've been great particularly for me who hasn't read a journal in a long time, going back reading journals and evaluating them has been a really nice … it's been a nice exercise and I feel like I've really improved at doing it over the past few months.*

*PT10: I really enjoyed it. It really was nice to get back to that critique, that kind of back to your academic head you know and reviewing the literature like that, it was great.*

Two-thirds of the physiotherapists (8) described how the fact that they were challenged by the CoP activities was an important part of their development and growth.

*PT01: I like being I suppose pushed out of my comfort zone a little bit which is why I did it.*

*PT 10: I think we've done more than I had expected in a short period of time and it was done at the right pace, it challenged people.*

*PT11: I've got a lot more from it than I would have thought personally … it's forced me to really sit down and spend time reviewing the literature and coming to my own conclusions. I got the most out of reviewing the journal club so far, it's been brilliant, and that's really a personal learning experience really.*

## 4. Discussion

This study is the first to describe the experiences of practising physiotherapists engaged in a CoP. Responses from the interviews indicate outcomes that are typical of a newly established CoP [6], that is, learning together through peer support, knowledge sharing through mutual interaction in the journal clubs and dissemination through the website, and knowledge creation, through development of new resources.

While Dannapfel et al. [11] showed that physiotherapists are highly intrinsically motivated towards EBP, a desire for peer support appeared to be a stronger motive for joining the CoP in this study. This may be a reflection of the fact that most of the participants were based in primary care settings, where they were isolated from regular access to other physiotherapists, but emphasises the potential value of a CoP as a tool for facilitating continuing professional development (CPD) and peer support for primary care clinicians. While the value of peer learning has been confirmed in undergraduate clinical education [12], there is limited evaluation of its role in the workplace setting. The physiotherapists in this study strongly emphasised the importance of connecting with peers in the CoP, both in terms of motivation to join and in gaining benefit from CoP activities jointly undertaken. How to achieve translation of research knowledge to inform clinical practice is an acknowledged concern raised across all health professions. A systematic review of knowledge translation interventions for rehabilitation professionals concluded that active, multicomponent interventions were successful in enhancing both knowledge and practice behaviours in physiotherapists [13]. Gunn and Goding [14] undertook a qualitative study of CPD among primary care physiotherapists, who reported a wide variety of CPD activities. Similar to our study, there was evidence of change in the therapist's clinical practice and internal perceptions, in particular confidence, as outcomes of their CPD. Physiotherapists in our study reported practice changes, with particular reference to how they educated patients, and explained the evidence behind their treatment choices, illustrating a knowledge translation process in action [15]. While the journal clubs were the most valued component of the CoP, participants also appreciated the resources that had been created through the CoP, for example, website and shoulder class protocol, which were transferable and sustainable products of the work that had been undertaken, and further spread the knowledge translation process to physiotherapists beyond the CoP.

The theme of personal development was closely aligned with the sense of being challenged for two-thirds of the participants. Some learning theories suggest that learning and growth happen most successfully just outside a person's “comfort zone,” as long as levels of anxiety are managed by providing appropriate supports [16]. A CoP can provide these supports through the contributions of peers, in addition to careful structuring of CoP activities by the CoP leader. It is noteworthy that although this is not the focus of the current study, just two physiotherapists mentioned the usefulness of the academic experience of the CoP leader in guiding the Shoulder CoP, suggesting that participants did not particularly emphasise her role in their CoP experience. Academic-clinical partnerships have been proposed as a way of improving knowledge translation, with Austin et al. [17] describing the positive impact of one such partnership on developing a successful journal club for physiotherapists. However, in our study, it appears that role of the academic as the CoP leader was not considered critical by participants and that a similar CoP could possibly be developed within a clinical or professional body setting among motivated individuals.

The barriers to involvement in the CoP discussed in this study reflect those commonly cited in the literature as barriers to undertaking evidence-based practice activities, these being time pressures and lack of research skills [2]. However, the participants in this study counterbalanced these barriers, mainly through their own motivation to participate, with a 70% attendance rate at CoP meetings. Members used time outside work to complete reading activities and improved their EBP skills with guidance from the CoP leader, peer support, and practice. During the time of the CoP project, Ireland's health services were undergoing severe resource challenges, staff shortages, and burgeoning waiting lists, with a concomitant difficulty in releasing clinical staff to participate in activities deemed to be nonclinical. Participation in meaningful and appropriate CPD to maintain quality of clinical care remains a priority to support physiotherapy practice. Gibbs [18] discusses the need to develop innovative means of providing CPD with no direct costs to the health service in such environments. Communities of Practice, which are participant driven and directly focused on clinical practice, provide such an innovative workplace-based CPD opportunity. We recommend the CoP model to physiotherapy managers, as significant benefits are gained in terms of EBP, patient care, and staff development, with excellent motivation from physiotherapists to engage with CoP activities.

The study had a few limitations. The research team was not independent of the CoP process, as it included the CoP leader and research assistant on the CoP project, both of whom undertook data analysis, with interviews conducted by the research assistant. This may have influenced the responses of participants in order to present the project in a positive light to those with whom they have been working. The sample was not complete, as four out of the sixteen CoP participants were unavailable for interview.

## 5. Conclusion

This study provides support for the use of a Community of Practice project to develop EBP for primary care physiotherapists. Physiotherapists valued the support and experience of peers in the CoP and described positive changes in their clinical practice as a result of CoP activities. Barriers to participation were overcome through intrinsic motivation from CoP members and appropriate organisation of the CoP.
